# Building the eye care team

**Published:** 2014

**Authors:** Thulasiraj Ravilla, Gnanasekaran Chinnathambi

**Affiliations:** Executive Director: Lions Aravind Institute of Community Ophthalmology, Aravind Eye Care System, Madurai, India; HR manager: Aravind Eye Care System, Madurai, India. gnana@aravind.org

**Figure:**
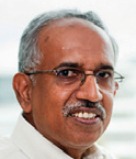
Thulasiraj Ravilla

**Figure:**
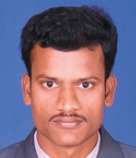
Gnanasekaran Chinnathambi

Eye care services are people intensive. They require the right people (competence), in the right numbers (capacity), in the right mix (team) with the right resources and processes (enabling conditions) to ensure effective and sustainable delivery of patient care.

To be effective, the team should have the right mix of ophthalmologists, ophthalmic assistants, administrative staff and support staff. It is important for each team member to be clear about what is expected of them and how their tasks relate to the overall purpose of the organisation that employs them. Building an effective workforce is a continuous journey. Inadequate staff numbers, lack of competency, and low motivation and productivity are common challenges. In our experience, however, systematically addressing all of the following areas contributes to success.

**Recruitment and selection.** In some settings, particularly in government programmes, there may be little choice in the selection of staff. However, where the eye care service has the flexibility to manage its own recruitment, there are two distinct steps. First, in order to get a pool of suitable candidates, each post should have a clear job description and be advertised appropriately with enough time for the candidates to respond. The selection process should probe not just the candidate's competency but also his or her motivation. The reality is that technical skills can be developed and improved overtime, but it is much more challenging to change fundamental beliefs, attitudes and behaviour. Thus the recruitment and selection process should ensure both competency and the right fit for the organisation's values, but with a greater emphasis on the values (which are reflected in the organisation's mission and vision).

**‘The recruitment process should ensure the right fit for the organisation's values’**

**Orientation (induction).** On joining the hospital, the new employee should undergo a well-designed orientation (induction) to help her or him to become familiar with the new surroundings. This should be led by the newcomer's designated line manager. The orientation should aim to deepen the new employee's understanding of the organisation and cover its aims, history, key members, operating model and values. It should also clarify the person's roles and responsibilities and how these relate to the organisation's mission and vision. The orientation lays the foundation for working together as a team.

**Figure:**
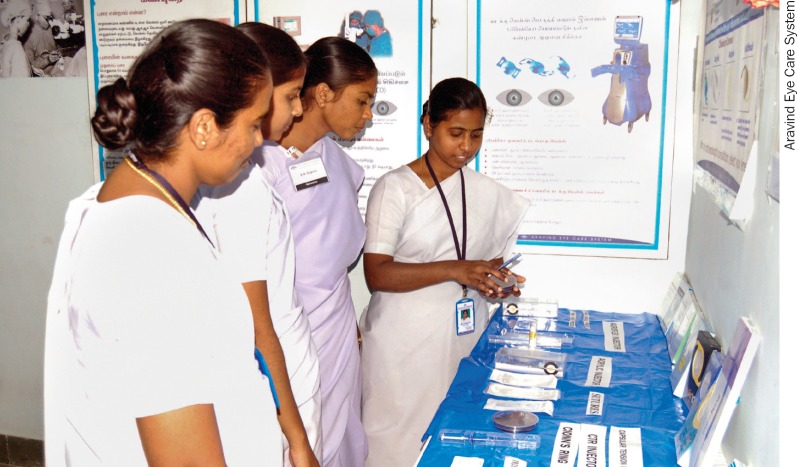
Operating theatre nurses are given an orientation session in the use of IOL injectors

**Enabling environment.** The hospital has to create an enabling environment for its employees by ensuring that they have the required resources (patients, equipment, supplies and support staff) and also the understanding and authority they need to take decisions and accomplish their tasks. For instance, the nurse in charge of the operating rooms should have the authority to requisition supplies as required, and the person in charge of transportation should have the authority to hire vehicles as needed. Such operational decisions should not require the approval of the medical director. Responsibilities can be made clear through sharing all job descriptions with members of the team, and be reinforced through regular team meetings – ideally once a week. Trends in eye care technology, changing patient expectations, as well as constraints relating to the availability of trained eye care professionals and supplies, should also be taken into account.

**Employee empowerment.** Team meetings should have a clear agenda and foster an open and democratic environment that encourages members to jointly address any difficulties, such as a temporary shortage of a particular consumable. Employees should also be encouraged to initiate new projects and share their ideas about how to improve patient care. Having a say in operational level decisions enhances people's engagement in their work and creates a culture of ownership among employees.

**Performance assessment and feedback (appraisal).** There should be a formal system for each employee and her or his line manager regularly to discuss the employee's performance (at least once a year). This discussion should be held in an open and non-threatening way, and should be recorded on a standard form. It should provide constructive feedback on performance and on any concerns. Together, the employee and line manager must draw up an action plan to upgrade skills and/or improve behaviour. The plan should include how best the organisation can support this process.

If an employee has serious problems of competence or behaviour, mentoring may be considered. An experienced staff member could be assigned to help the employee resolve problems relating to work life, or to provide support when there are problems in her or his personal life.

**Career development and training.** When employees show motivation and ability, they should be given additional responsibilities (with appropriate training) if possible. This could be an opportunity to lead a new activity and improve their leadership skills. The organisation should endeavour to have a well-defined career path for all types of employees, as this builds people's aspirations towards the next level in their career. When an employee shows ability and a desire to move to a new position within the organisation, it is good to be supportive to the extent it is practical. This could both help the employee (self-development) and retain her/his skills. The organisation should periodically conduct training needs assessments for all employees and design training programmes based on the needs identified.

**Pay and benefits.** This is one of the most sensitive aspect of employment. To an employee, the salary is the indicator of how they are valued in the organisation. More often than not, dissatisfaction with pay is the reason people leave an organisation. Where possible, try to ensure that salaries are competitive (compared to those offered by similar organisations) and represent fair market rate. Internally, employees’ pay and benefits should be comparable to that of others within the organisation doing similar jobs. If pay rates are set externally, however, whether by the government or other bodies (e.g churches), it may not be possible to do very much.

**Retention and welfare.** Employee retention should be a major concern for any organisation. Individuals, once well trained and effective, may opt to move for better prospects. In that case, the line manager or leadership team may try to convince the employee to stay by offering a new challenge, such as additional responsibilities which carry extra benefits. If the employee is determined to leave, a formal exit interview is useful to inform management about her or his experiences within the organisation and what might be improved.

The organisation has to be concerned about employees’ physical, mental, social and spiritual wellbeing. Health insurance, scholarships for career development or child care are some of the welfare measures that can motivate people to stay. Social events and competitions to demonstrate talent can also encourage creativity and foster team spirit.

The performance of a hospital is a reflection of the quality of the people who work there. It is therefore essential to recruit and retain employees with the right attitude, competence and potential.

**FROM THE FIELD: Retaining the eye team: top tips from Kenya**

**Figure:**
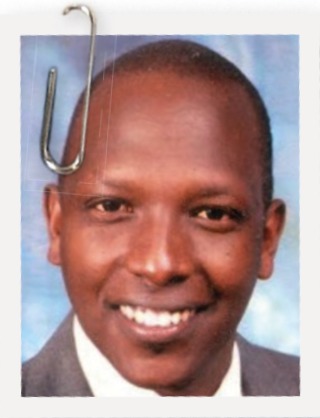

**1 Kitale District Hospital Eye Unit**

**Hillary Rono: Ophthalmologist**

- At our hospital, eye care workers undergo regular refresher training in surgical and clinical skills, instrument care and good ‘customer’ relations. This is done through attachments at high-volume hospitals, an annual workshop on instrument care and a programme in which they are mentored by an ophthalmologist. In the mentorship programme, existing talents and skills are identified and developed and staff are encouraged to acquire new skills. This ensures that high quality service is maintained.
- Personnel are given on-the-job training in other tasks. Support workers (with no clinical background, e.g. people who provide clerical, cleaning, equipment maintenance and financial services) are trained to test visual acuity, dilate eyes and to assist in theatre. Skilled eye care workers (ophthalmologists, clinical officers and ophthalmic nurses) are familiarised with operations management and bookkeeping.
- Performance targets are set at the beginning of every year by the whole eye care team. These targets form the basis of performance assessment and feedback (appraisals).
- Keeping staff motivated over a long period of time is a challenge, as the needs of individuals change over time. Within the eye unit, we offer supportive supervision (a respectful and non-authoritarian approach that involves joint problem solving^1^) and appraise staff every quarter (i.e. we assess their performance and provide feedback). We also offer opportunities to participate in outreach programmes and in quality improvement programmes (based on feedback from cataract surgical outcome monitoring). We encourage and support staff to attend relevant continuous professional development workshops and training, and to collaborate on research projects with other institutions. The annual appraisal of the eye unit performance within the hospital also helps to motivate staff.

Further reading1World Health Organization. Supportive supervision (training module). Visit www.who.int/immunization/documents/MLM_module4.pdf

**2 Kwale District Eye Centre**

**Figure:**
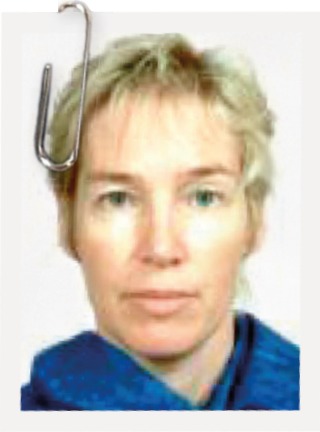

**Helen Roberts: Medical Director**

- At our hospital, staff are encouraged to take the leave they are entitled to.
- There is a sensible sickness policy and staff are well looked after if sick.
- Punctuality is respected, both on arrival to work and completing the day. Our staff register is kept in an open plan office, so staff sign in and out in my presence. This is a good opportunity to meet and greet each staff member and to discuss the day ahead, or the day past, or to simply acknowledge them.
- Staff have a nice uniform and clearly written name badges. Once a name badge is in place, everyone knows who they are and what their job is. Any visitor can pinpoint a staff member as having served them, so it encourages a sense of responsibility.
- Staff have regular tea breaks. We have found that it is more difficult for tired, thirsty staff to give good customer care.
- We acknowledge and talk about teamwork and emphasise its importance at meetings and in general.
- Although meetings take time, they are important for communication and motivation. It helps to know that we are aware of each other's challenges and share responsibility for addressing them. As medical director, I check the minutes of each department's regular meetings to ensure they are taking place.
- Moving people around departments isn't always comfortable, but it helps our team members to understand how the other departments work. If it is not appropriate for someone to work in a department, we make sure they visit (e.g. the adminstators visiting outreach activities).
- We have an informal social event for the team at least once a year.
